# Cementless augmented versus cemented Dual Mobility cups: medium-term outcome of case series with a control group

**DOI:** 10.1186/s12891-023-06204-4

**Published:** 2023-02-06

**Authors:** Ayman Ebied, Ahmed Ali Ebied, Ismail Tawfeek Badr, Mostafa Affara, Sameh Marie

**Affiliations:** Department of Orthopedic Surgery, Menoufia University Faculty of Medicine, Shebin El-Kom, 32511 Menoufia, Egypt

**Keywords:** Coptos TH, KE ring, Impaction graft, Acetabular defects, Revision THA, Dislocation

## Abstract

**Background:**

Post-operative dislocation and reconstruction of acetabular defects are two challenging topics in revision Total Hip Arthroplasty (rTHA). Cemented Dual Mobility (DM) cups on top of Kerboull Cross and bone graft have been successfully employed to overcome these challenges. The cementless augmented DM cups were recently introduced. In this study medium term results of the augmented cementless DM Coptos cups are reported and compared to the established technique of cemented DM cups and Kerboull plate.

**Material and methods:**

This is a retrospective analysis of data collected on patients who received rTHA using DM cups in the period between June 2015 and September 2020. Two groups of patients were identified. The first group received cementless augmented DM-cups (NOVAE® Coptos TH–SERF) (Coptos TH cup group). The second comparable group who had Kerboull ring (KE ring group) and cemented DM cups (NOVAE® STICK). Demographic data, surgical technique, functional and radiological outcome as well as complications during the follow-up visits are reported.

**Results:**

Forty-two patients with a mean age at the time of revision 48.8 ± 13.6 years. 29 patients received Coptos TH DM-cup, while 13 patients had Bone Graft (BG), KE ring and cemented DM cups for acetabular reconstruction. Acetabular defects were Paprosky types IIB and IIC in 31 patients and IIIA and B in 11 patients. The follow-up was 52.8 ± 21 months (mean ± STD); and the mean Harris Hip Score (HHS) at last visit was 91 ± 5. Good stability of all cups was reported. Full integration of the impaction graft was observed in 94% of the Coptos and 92% of the KE groups. One of the Coptos cups was readjusted and one case of single dislocation was recorded in the KE group. None of the DM cups in both groups was revised or awaiting revision.

**Conclusion:**

Coptos TH cups achieve similar results to the cemented DM on KE ring at the medium term but long term outcome remains to be seen.

## Introduction

Dual Mobility (DM) THA has proven its value in reducing postoperative instability, especially in patients with a higher risk of dislocation [1]. In comparison to patients who received 40 mm head diameter, the DM construct showed a lower risk of revision for dislocation, re-revision, and readmission to theatre [2] Hence, numerous surgeons prefer to use the DM construct in revision THR regardless of the underlying cause for revision [3].

In presence of an acetabular bone defect, Kerboull plate and cemented DM cups are commonly employed [4]. Wegrzyn et al. reported 994 revisions THA at a mean follow-up of 7.3 years and an incidence of dislocation of 1.5% and 2 cases of Intra-prosthetic Dislocation (IPD) of 0.2% [4]. Similarly, Simian et al. reported a 99% survival at 5 years for 71 patients who had revision THA using DM articulation while postoperative dislocation was seen in one patient [5]. Schnieder et al. reviewed 96 rTHA using cemented DM cups and different acetabular reconstruction cages. The survival of cups at 8 years was 95.6% while it was 99.3% if acetabular exchange for aseptic loosening was taken as the endpoint [6].

Modern designs of modular DM cups allow surgeons to use a standard cementless shell with screws, an inner polished metal liner that articulates with a high cross-linked polyethylene (HXLPE) mobile insert. Metal debris at the interface between inner liner and outer shell with increased serum metal ion level is a concern that needs further surveillance [7]. Additionally, IPD in the early postoperative period was reported [8].

Meanwhile, parallel developments have been added to the cementless DM Monoblock cup designs to allow for extra-articular fixation using flanges and screws, e.g., the Coptos TH cup (NOVAE® Coptos TH – SERF). These cups may facilitate cup fixation in cases of deficient acetabular bone [9, 10].

The outcome of the augmented cementless DM cups has only been reported once on a group of complex primary and revision THA [11]. In this study, the medium-term outcome of Coptos TH – cups will be presented and compared to the established technique of cemented DM cups on the Kerboull Cross plate (KE ring). The tested hypothesis is whether these recently introduced cementless Coptos cups can produce at least a similar rate of survival at the medium term.

## Material and methods

In the period from June 2015 to September 2020, 307 hips (300 patients) received dual mobility THA at our unit.

### Selection criteria

**Fig. 1:**
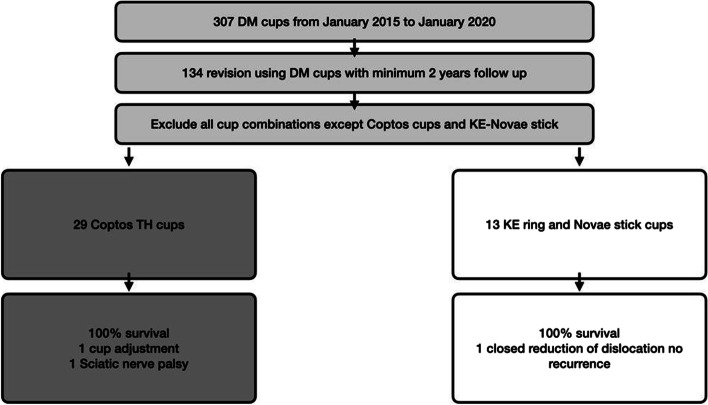
Flowchart of the studied groups

The inclusion criteria were a dual mobility articulation used in revision THA with a minimum 2-years follow-up. This left us with 134 rTHA who received DM articulations. Different cups and construct combinations were excluded from this analysis except for two categories, the first is NOVAE® Coptos TH–SERF (Group I). The second is cemented DM cups (NOVAE® Stick—SERF) on top of the Kerboull Cross plate (KE ring—SERF) (Group II). Thus, 42 hips (in 42 patients) were included in the final analysis, 29 in Group I and 13 in Group II (Fig. 1).

### Study design

This is a retrospective analysis of prospectively collected data. All patients were evaluated clinically using the Harris hip score (HHS) [12] preoperatively and at the latest follow-up visit. At the final review, 1 patient from group II had deceased after > 2 years of follow-up. 5 patients could not attend for clinical evaluation. However, recent x-rays were acquired and phone interviews were conducted.

Demographic data, including age at the time of surgery, gender, indications for revision, types of revision (acetabular component or both femoral and acetabular), the use of impaction allograft, complications, and/or readmission to theatre were all recorded.

### Radiographic evaluation

**Fig. 2:**
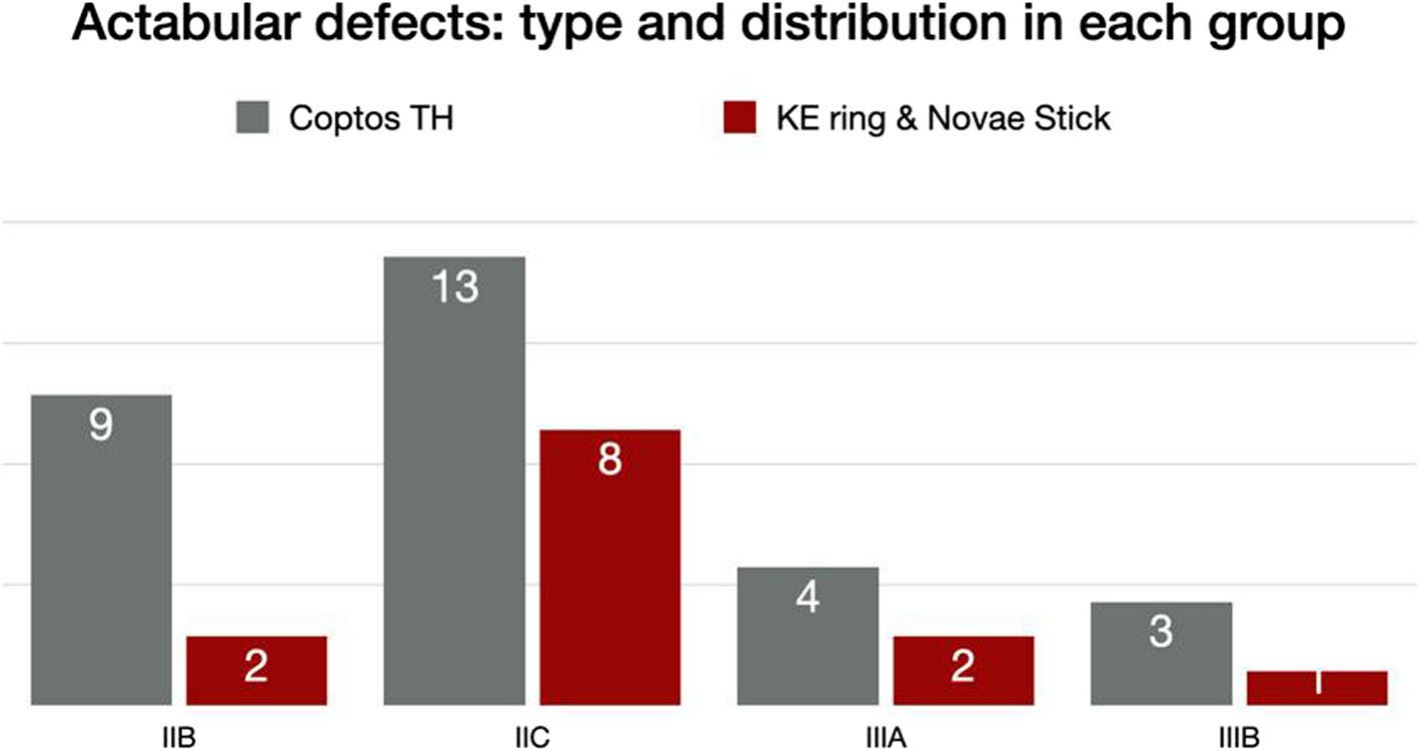
Acetabular defects: types and distribution in each group

Acetabular bone defects were analyzed preoperatively using Paprosky classification [13, 14] (Fig. 2). Standard pelvic x-rays were evaluated for cup position, abduction angle, and cup-bone interface for cementless or cement mantle integrity in cemented cups. The stability of all cups was assessed by comparing the 6 months post-operative to the most recent x-rays. The presence of progressive radiolucent lines around the cups or the screws used for extra-articular fixation in both groups was recorded. Incorporation of the bone allograft was evaluated according to the Conn et al. criteria. Full graft incorporation was defined as a similar density to the allograft and patient bone with continuous trabecular arrangement throughout the graft-host bone junction [15].

Underlying etiologies for revision surgeries were aseptic and septic loosening of previous implants, dislocation, and acetabular erosion over hemiarthroplasties Table 1.

**Table 1:** Epidemiologic data, clinical outcome and indications for revision surgery

	Coptos cup group(no = 29)	KE ring group(no = 13)	Total(no = 42)
Age	47.83 ± 14.6	50.85 ± 11.3	48.76 ± 13.6
Gender			
Female	14	6	20
Male	15	7	22
Side			
Right	13	6	20
Left	15	7	22
Follow up (Months)	54.2 ± 20.6	49.6 ± 23	52.8 ± 21
Preoperative HHS	67.7 ± 8.57	58.4 ± 4.35	64.8 ± 8.6
Postoperative HHS	91.8 ± 5.9	92.2 ± 5.4	91.9 ± 5.68
Size of acetabular implants	58.8 ± 5.56	55.54 ± 3.6(48.7 ± 3.3 cemented DM CUPS)	
Indications for revisions	Revision for aseptic loosening of cemented/cementless THR (15 cases)	Revision for aseptic loosening of cemented/cementless THR (6 cases)	21
	Single Stage Revision PJI (4 cases)	Single Stage Revision PJI (1 case)	5
	Aseptic loosening in Bipolar Hemiarthroplasty with acetabular defect (5 cases)	Aseptic loosening in Bipolar Hemiarthroplasty with acetabular defect (4 cases)	9
	Aseptic loosening of THA Originally performed following failed fixation of hip fractures (5 cases)	Aseptic loosening of THA previously originally performed following failed fixation of hip fractures (2 cases)	7

### Surgical technique

**Fig. 3:**
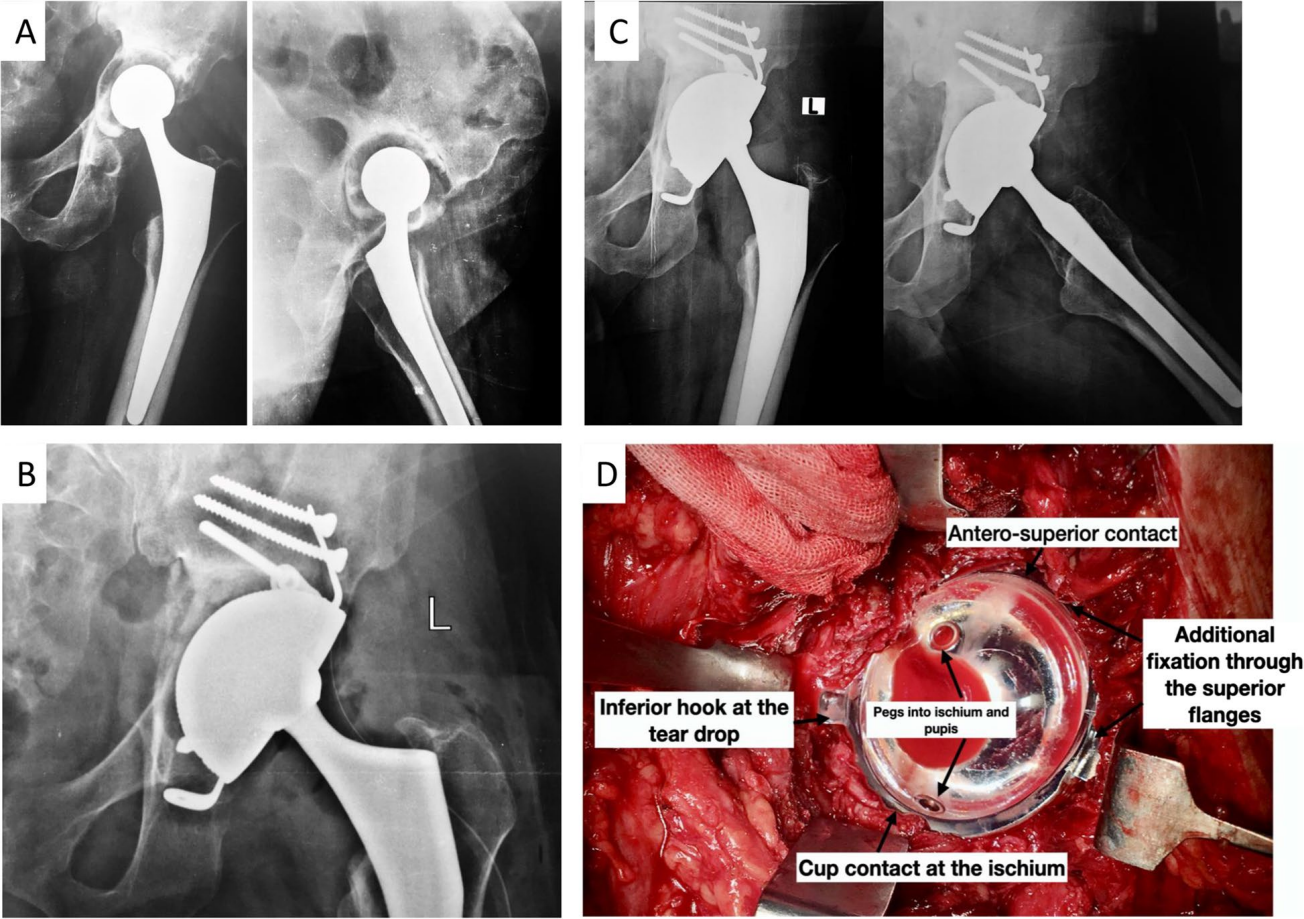
**A** Preoperative x-ray of a loose cemented cup with proximal migration > 20 mm and medial defect Paprosky IIIB. **B** x-ray at 3 months posts operative showing cup position and medial graft. **C** 3 years post-operative with cup stability, restoration of the hip center of rotation, and graft incorporation. **D** Intraoperative photo demonstrating Coptos cups rim contact in addition to intraarticular and extra-articular additional fixation

The surgery was performed through the posterior approach ± Extended Trochanteric Osteotomy (ETO). Having removed the loose implant, the severity of the acetabular bone defect was re-assessed. Implant choice was based on the integrity of the remaining acetabulum bones, particularly the ischial and anterosuperior walls. When a stable rim contact was achievable between the hemispherical cup and the above two areas, the Coptos TH cup (NOVAE® product line SERF, Décines-Charpieu, France) was implanted with additional extra-articular fixation using screws through the superior flanges and hydroxyapatite (HA) coated pegs into the ischium and pubis (Fig. 3). Fresh frozen allograft were impacted in the medial defects (Fig. 3).

**Fig. 4:**
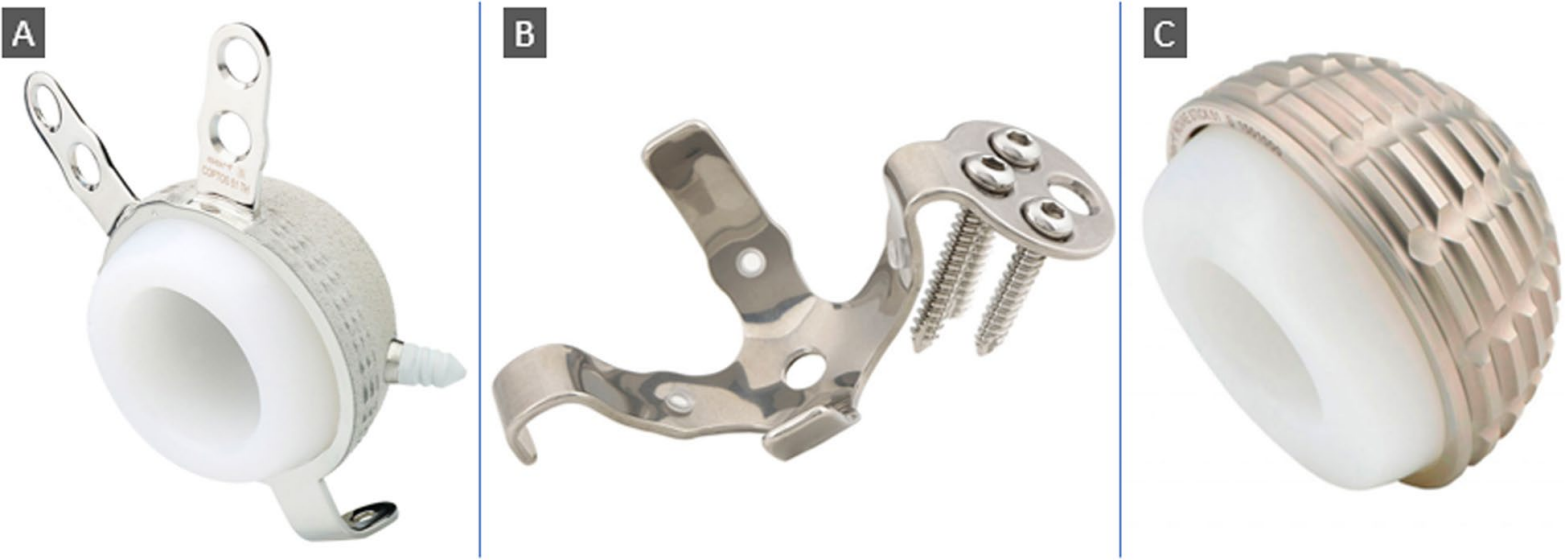
**A** The Coptos cup is HA-coated and has an acetabular hook, two iliac flanges, and HA-coated pegs to be impacted in the ischium and pubis. **B** The KE ring. **C** Novae stick cemented cup. (Photos were provided by the manufacturer, and an authorization request was taken)

**Fig. 5:**
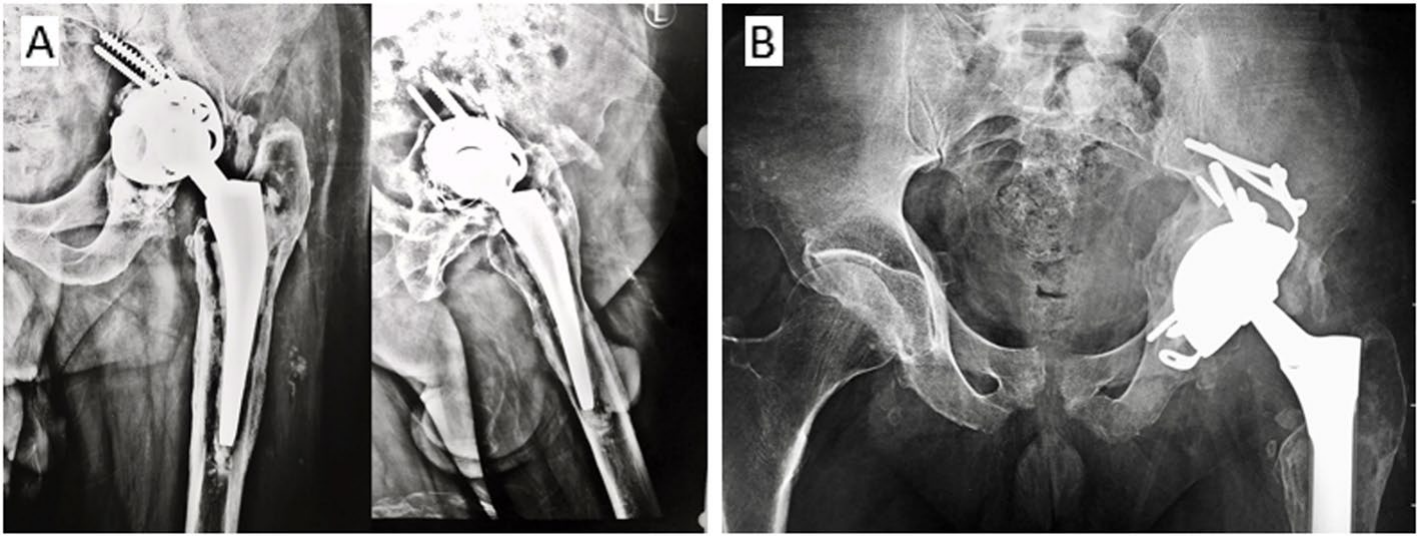
**A** Preoperative x-ray for a loose cemented cup and Muller ring from an earlier revision THA Paprosky IIIB bone defect (**B**) 4 years postoperative x-ray of a stable Coptos cup and incorporated bone graft that was impacted in the medial defect

The augmented Coptos TH DM cup is a third-generation DM articulation with press-fit and supplemental fixation. It is made of 316L stainless steel that is covered by plasma-sprayed titanium and HA. Supplemental fixation comprises an acetabular hook, two superior flanges, and two acetabular pegs (Figs. 3, 4 and 5).

**Fig. 6:**
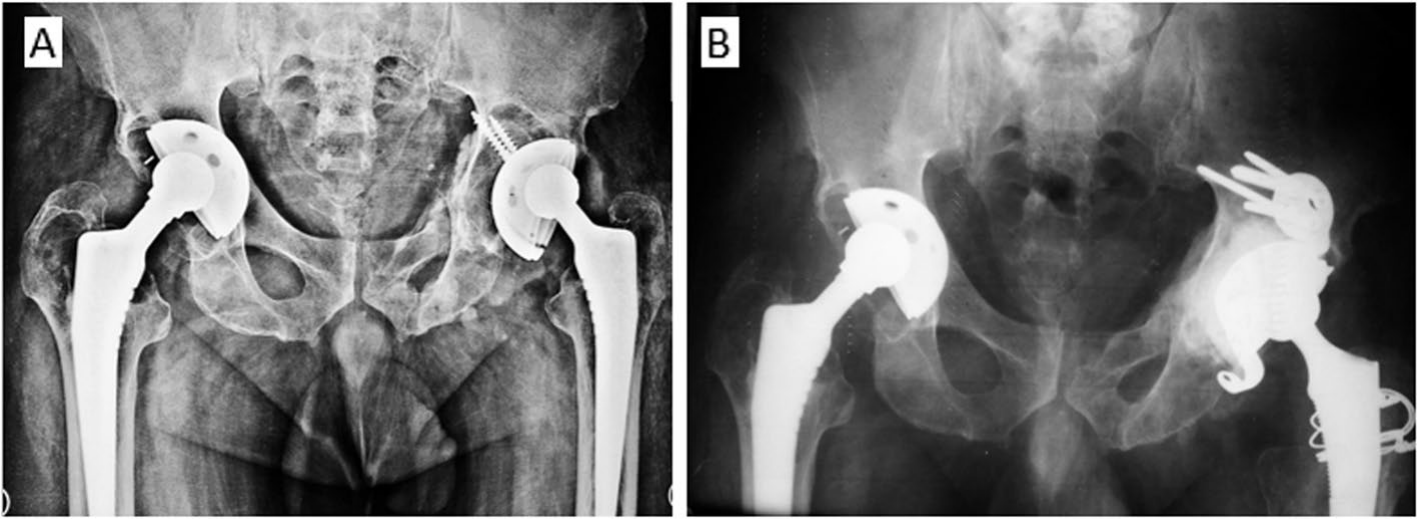
**A** preoperative x-rays for loose left acetabular cup with massive osteolysis Paprosky Grade IIIB and fracture pubic rami that indicated impaction graft and KE ring. **B** postoperative x rays with the full incorporation of the impaction graft, healing of pelvic fractures and stable cemented Novae stick cup

The indication for implanting a KE ring and NOVAE® stick cup were severely osteoporotic and non-supportive walls for the primary stability of a hemispherical cup to be achieved. The four arms of the KE ring were inserted within the acetabulum boundaries supported by the host or graft bone. Four cortical screws of 4.5 mm were directed through the plate towards the sacroiliac joint to secure the position of the KE ring. Cement pressurization and insertion of a NOVAE® STICK cup completed the acetabular reconstruction (Figs. 4 and 6).

The acetabular defects were Paprosky type IIB and IIC in thirty-one patients and Paprosky type III in eleven patients (6 IIIA and 5 IIIB) Figure II. Cavitary acetabular defects were grafted with impaction of fresh frozen bone allograft (8–10 mm cancellous bone chips obtained from femoral heads mixed with Vancomycin powder, 4 g per head).

All patients were allowed touch weight-bearing (WB) from the first post-operative day, which progressed from partial to full WB at 3 months.

Clinical evaluation using the HHS in addition to radiological assessments were performed postoperatively at 3 months, 6 months, then annually afterward.

Two endpoints for the study were considered in the final evaluation. The first is re-admission to theatre for any reason and the second is cup revision.

### Statistical analysis

IBM SPSS version 25.0 (SPSS Inc., Armonk, NY) was used for the statistical analysis of data. Categorical variables were compared using the chi-square or Fisher’s exact tests, while continuous variables were compared using the student’s t-test or Mann–Whitney U test, and the mean and standard deviation (SD) were calculated for descriptive variables. Statistical significance was set at a *P*-value < 0.05.

## Results

Case series of forty-two patients (22 female and 20 males,22 left and 20 right) were included in the final analysis of this study. 29 patients (69%) had Coptos TH DM cups, while 13 patients (31%) had KE ring and cemented NOVAE® STICK DM cups for reconstruction. The mean age at the time of the revision was 48.8 ± 13.6 years.

No statistically significant difference between both groups regarding age, gender, side, acetabular defects, follow-up period, preoperative HHS or postoperative HHS was found.

Demographic data of both groups and Indications for revision surgery are reported in Table 1. The previous surgeries in these patients were primary THA, first stage revision with spacer implantation, previous revision surgery for dislocation, infection or cup loosening, reduction for hip dislocation, and previously performed primary THA following failed fixation of hip fractures. No statistical difference was seen in regard to the previous surgery or their numbers between both groups (*p* = 0.528).

Both acetabular and femoral components were revised in thirty-seven hips and only acetabular component revision in five hips (21%).

Impaction graft was used in all patients (13/13) in the KE ring group while it was used in 17/29 (58%) in the Coptos cup group.

In the KE ring group, the mean diameter of the Kerboull cross-plate was 55 mm (range: 50–60 mm), with a mean diameter of the cemented dual-mobility cups of 48 mm (range: 45–55 mm), while the mean diameter of the implanted Coptos cup was 58 mm (range:47- 65 mm). (Figs. 3, 5 and 6).

The mean follow-up was 52.8 ± 21 months (range: 24–91 months). In the Coptos group a significant improvement in the HHS was observed from preoperative 64 ± 8 to postoperative 91 ± 5 (mean ± STD) *p*-value˂0.001. Similar improvement of the HHS was recorded for patients in the KE ring group from 60 ± 8 preoperative to 87 ± 5 postoperative (*P* < 0.001). No difference between the groups was seen (*P* < 0.1). Two patients in the KE ring group had functional limitations due to other joints pathology (Fig. 6) and are scheduled for revision surgery to the contralateral hip.

### Radiographic evaluation

Comparison of x-rays taken 3 weeks postoperatively to those obtained at the latest follow up was made. In the Coptos group, all cups were stable with good osteointegration at the cup host bone contact areas. No change in cup abduction angle was observed in all Coptos cups except one. Five degrees of change in cup abduction angle in one cup was seen at 6 weeks in comparison to the immediate post operative, yet remained stable since. No radiolucent lines were observed around the pegs or flange screws. Full incorporation with trabecular bone formation was observed for the impaction graft used in 16/17 patients who received the graft (94%) (Fig. 3).

Stable cemented DM (Novae stick) cups were seen in all hips on the KE ring group. Full incorporation of the BG was observed in 12/13 patients (92%) in this group. The technique of grafting and stable fixation of the KE ring using four screws into the ilium has even helped healing of insufficiency fractures in the pubic rami and medial acetabular walls (Fig. 6).

Three complications were reported. The first was a single postoperative dislocation for a patient in the KE ring group who had his revision for recurrent instability. Closed reduction was possible without further episodes of instability. The second complication was readmission for adjusting a Coptos cup after 6 weeks from surgery Fig. 3. The same patient had partial sciatic nerve palsy in the distribution of the common peroneal nerve. Full recovery of nerve function observed after 6 months. It is worth noting that IPD was not seen in any of the patients in both groups.

Femoral stems were revised in 37 cases. Straight monoblock long revision Wagner stems 20/37 and anatomic fully HA-coated stems with distal locking (Sagitta revision system SERF®) 17/37 were employed in these revisions.

When re-admission to theatre was taken as an end points, the success rate of the Coptos group was 97% and the KE group 92%. Meanwhile, 100% survival of both types of DM cups was reported at the latest follow up.

## Discussion

An important finding in this study was the reported success of the revision cementless Coptos TH cup when compared to the established technique of the KE ring and Novae stick cemented cup at a mean follow-up of 52 months. Additionally, the use of impaction graft with a cementless implant was found to have a high rate of incorporation and improves bone stock in case future revision is required. It is important to note here that rim contact with host viable bone and primary stability of the cementless cup (Figs. 3 and 5) were the determining factors in choosing Coptos TH versus cemented NOVAE® STICK implant.

When compared to a large head diameter 40 mm on a fixed liner DM articulation was found superior in reducing postoperative dislocation, re-revision, and all causes for re-operation [2]. The reported incidence of dislocation in primary THA ranges from 0.4% to 5.8% [2, 16, 17]; while in revision higher incidences of postoperative instability from 14 to 20% were reported [18]. Philippot et al. studied 438 hips employing DM cups and reported excellent survivorship (96%) with no early or late instability after 15 years of follow-up [19]. A systematic review by De Martino et al. revealed a low incidence of dislocation in revision THA of 1.3% when DM cups were used [20]. In this series of 42 revisions, one case of postoperative dislocation was reported 2%. Hence, the question that should be raised is, which DM cup can help revision patients with acetabular bone defects to have a lower risk of dislocation and longer survival of their implants?

Prudhon et al. presented the early results of using cementless DM cups in 79 revision THA [9]. In their study, 68 standard DM cups were used for grades I&II Paprosky defects while special revision cups were employed in 11 hips that had Paprosky grade III defects. They reported one case of dislocation and early mechanical loosening in 2 hips (2.7%) at 2 years.

In this study, 45% of hips (19 out of 42) had Paprosky type IIC and III (A&B) acetabular defects with proximal migration of the center of hip rotation > 20 mm, yet primary stability of the hemispherical cup was achievable with the aid of the extra-articular fixation flanges and intra-cup pegs. At the medium-term stable cup position, bone ingrowth and stability were observed in all cups.

An important observation in this study is the lower rate of complication, especially when compared to an alternative like the Constrained Acetabular Liner (CAL). A recent systematic review for the CAL revealed a high complication rate of > 22% in cases of primary and rTHA. CAL were associated with high incidence of dislocation, infection and aseptic loosening [21]. It is therefore better kept as a salvage implant to cases with complete loss of the abductor mechanism function.

Using bone grafts with cementless cups is debatable, with the fear of reducing the cup to host bone contact [22]. However, the usually quoted percentage of minimum 50% contact between the cup and host bone was related to bulk grafts that have a different mode of incorporation and substitution from the fresh frozen chips impacted in 58% of Coptos TH cup cases and revealed full incorporation in 94% of the hips within 24 months. The value of the bone graft was to aid in cup stability, a carrier for the antibiotics, and restore bone stock in case future revision become necessary [23–26].

Cemented all poly cups on top of impaction bone graft IBG is a well established technique with or without the use of metal mesh [27]. Recent review article confirmed a high success rate with aseptic loosening of acetabular component in only 3% at mean follow-up of 6.1 years (range 3–10 years) [28]. While there is a high agreement on the success of IBG in moderate acetabular defects doubts exist on the long term survival of this technique in the face of massive defects type III A & B Paprosky especially when more than one metal mesh is required [29].

This is a young group of patients 48.8 ± 13.6 years (mean ± SD). Many of these patients had their original arthroplasty to salvage complicated hip trauma. As many of them are relatively young with high demand to regain good function and range of movement surgeons were encouraged to use the cementless DM articulation. Additionally, bone graft was used to help restoring the bone stock that may be needed in future revisions. As they are a young group of patients longer term follow up will be necessarily reported.

Results of this study support initial report on the success of Coptos TH cup [11] in revision cases with deficient acetabular bone stock. It is worth noting that Coptos cup was equally successful in patients with mild defects type IIB (9 hips) in comparison to the more severe defects IIC and above (20 hips). Press fit contact between the cementless hemispherical cups and host bone can usually be achieved in milder acetabular defects with less demand on the extra-articular fixation.

Modern designs of the modular DM cups allow for screw fixation and surgeon familiarity with shell application. Both are attractive options in revision surgery, but not without the added risk of non-similar metal contact at the liner-shell interface. Reports of increased metal ion levels besides early IPD were reported [7, 8, 30]. IPD was not observed in this series.

Kerboull plate and cemented DM cup is a well-established technique for acetabular reconstruction in revision THA [31]. Low rates of dislocation and survival of implants in the medium term were reported [1]. In this study, using the KE ring and Novae stick cup, comparable results were achieved. Restoration of the hip center of rotation and bone graft incorporation were recorded in 92% of the cases.

The weakness of this study could be related to the lack of randomization between the groups and the small number of patients. However, it is the first publication, to our knowledge, on the outcome of the Coptos TH cup outside the designing institute [11]. This study is presenting results of revision THA on patients with severe acetabular defects (Paprosky IIC, IIIA, and IIIB) that needed bone graft in contrast to the previous article that included complex primary THAs. Hence, it was important to present these data that focus on revisions for massive acetabular defects.

## Conclusion

DM articulation has a high rate of stability with a low risk of postoperative dislocation in revision THA. The development in the design of monoblock cementless DM cup allows reconstruction of massive acetabular defects faced in revision surgery and achieves comparable results to the KE ring and cemented cup technique at the medium term. Coptos TH cups have promising results even in a challenging revision, but long-term results should be reported.

## Data Availability

Available on request, the corresponding author is the responsible for data availability ( I B).
